# Calibration of low-cost sensor data for ambient PM_2.5_ monitoring across urban and rural settings in South Central Uganda

**DOI:** 10.1016/j.apr.2025.102580

**Published:** 2025-06-24

**Authors:** Sophia Le, William Checkley, Lauren Dudley, Joseph Ssuuna, Anthony Ndyanabo, Engineer Bainomugisha, Joel Ssematimba, Richard Sserunjogi, Deo Okedi, Deo Okure, Joseph Kagaayi, Larry Chang, Kirsten Koehler, Laura Nicolaou

**Affiliations:** a Department of Environmental Health and Engineering, Bloomberg School of Public Health, Johns Hopkins University, Baltimore, 21205, MD, USA; b Department of International Health, Bloomberg School of Public Health, Johns Hopkins University, Baltimore, 21205, MD, USA; c Rakai Health Sciences Program, Kalisizo, Uganda; d AirQo, Department of Computer Science, Makerere University, Kampala, Uganda; e Division of Pulmonary and Critical Care, School of Medicine, Johns Hopkins University, Baltimore, 21287, MD, USA; f Division of Infectious Diseases, School of Medicine, Johns Hopkins University, Baltimore, 21827, MD, USA; g Center for Global Non-Communicable Disease Research and Training, Johns Hopkins University, Baltimore, 21287, MD, USA

## Abstract

Air pollution is a leading risk factor for the global burden of disease. There are limited data from Sub-Saharan Africa (SSA). This study addresses significant data gaps in air quality monitoring in South-Central Uganda. Using a near-reference grade monitor and a network of 27 low-cost air quality monitors in the rural Rakai region and urban cities of Masaka and Kampala, we developed a locally tailored calibration model for ambient PM_2.5_ concentrations. Using calibrated data across all low-cost monitors, we then examined spatiotemporal trends in ambient PM_2.5_ concentrations. The calibration model, which adjusts for relative humidity, demonstrated robust performance with an R^2^ of 0.92, a root mean squared error (RMSE) of 3.83 µg/m^3^ and bias of −0.39 µg/m^3^ compared to an RMSE of 20.37 µg/m^3^ and bias of 15.96 µg/m^3^ with the raw data. Mean PM_2.5_ concentrations during the dry and wet seasons were 22.1 µg/m^3^ and 12.7 µg/m^3^ in rural areas, and 37.7 µg/m^3^ and 23.2 µg/m^3^ in urban areas, respectively. We observed diurnal variations, with PM_2.5_ levels peaking in the early morning (6 a.m.–9 a.m.) and late evening (6 p.m.–10 p.m.), correlating with peak traffic hours. Low-cost sensors can enhance air quality research in regions like South-Central Uganda that lack air pollution data. Calibration tailored to local conditions can improve measurement accuracy of ambient PM_2.5_ concentrations.

## 1. Introduction

The burden of disease from air pollution in sub-Saharan Africa (SSA) is poorly characterized due to the region’s lack of air quality monitoring. A recent systematic review reported that annual concentrations of fine particulate matter (PM_2.5_; particulate matter with an aerodynamic diameter of 2.5 µm and smaller) in SSA range from 19 µg/m^3^ to 170 µg/m^3^, far exceeding the World Health Organization’s recommended threshold of 5 µg/m^3^ (Katoto et al., 2019). However, air pollution data were only available from 18 out of the 47 countries in SSA. The geographic distribution of these studies varied significantly, with six studies each from Western and Eastern Africa, four from Southern Africa, and just one from Central Africa. Data collection for these studies occurred in the capital or major cities, and often for short durations. This limited scope highlights the challenges in obtaining comprehensive air quality data across the continent.

In contrast, regions such as North America, Europe, China, and India have extensive air quality monitoring networks and a wealth of research that provides a detailed understanding of air pollution sources, concentrations, and trends (Brauer et al., 2016; Cohen et al., 2017; Murray et al., 2020; Nicolaou and Checkley, 2021). Local environmental and socio-economic conditions in SSA, which differ significantly from those in high-income countries, worsen air quality and complicate efforts to study and monitor air pollution levels (Amegah and Agyei-Mensah, 2017; Kansiime et al., 2022; Li et al., 2022; Sepadi and Nkosi, 2022; World Health Organization, 2018). Rapid population growth and urbanization has led to increasing emissions from vehicles, industries, and residential sources (Amegah and Agyei-Mensah, 2017; Li et al., 2022). Compounding this problem, high-density urban settlements in SSA often lack adequate infrastructure and regulatory frameworks for air quality management (Borge et al., 2023). Frequent dust storms, particularly in the Sahel region, contribute significantly to worse ambient PM_2.5_ concentrations (Ginoux et al., 2012). The variability and intensity of dust storms can lead to sporadic spikes in PM_2.5_ that are difficult to predict and monitor consistently (Ginoux et al., 2012). Moreover, the prevalent use of biomass for cooking, which is common in both urban and rural areas, results in substantial indoor and outdoor air pollution (Bonjour et al., 2013; Gordon et al., 2023; Kansiime et al., 2022).

Despite advances with small-scale, low-cost monitoring efforts, research into the spatial and temporal variations of ambient PM_2.5_ in SSA remains limited. Several studies in East Africa have used low-cost monitors to collect hourly PM_2.5_ data over short periods (Abera et al., 2020; Awokola et al., 2022; Kinney et al., 2011; Pope et al., 2018; Singh et al., 2021), demonstrating the feasibility of low-cost monitoring. However, these studies also revealed significant limitations in scope, duration, and coverage, often focusing on specific urban centers without capturing the broader spatial and temporal variations. Similar challenges are observed in studies from other regions within SSA, such as Southern and Western Africa, where monitoring efforts are sporadic and lack the long-term, continuous data required to fully understand the variability of ambient PM_2.5_ across different settings (Naiker et al., 2012; Okello et al., 2023; Petkova et al., 2013). These constraints highlight the need for more extensive and systematic air quality monitoring across SSA.

In Uganda, most air quality studies have been concentrated in major urban centers like Kampala, the capital and largest city of Uganda. These studies have identified ambient PM_2.5_ concentrations often exceeding 100 µg/m^3^, attributable to traffic emissions, industrial activities, and biomass burning for cooking (Awokola et al., 2022; Coker et al., 2021; Okure et al., 2022; Schwander et al., 2014; Singh et al., 2021). The AirQo project, led by Makerere University, has made significant strides in addressing data gaps by deploying over 80 low-cost air quality sensors across Uganda, with a significant proportion in Kampala (Bainomugisha et al., 2023a, 2023b, 2024). However, there are limited efforts to monitor air quality outside of Kampala, revealing a disparity in data availability between urban and rural regions. Rural areas and smaller urban centers are still significantly underrepresented in air quality research, leading to substantial data gaps that hinder comprehensive assessments of air pollution impacts across the country.

In response to these limitations, we deployed a network of low-cost air quality monitors to bridge the data gaps in the rural Rakai region and urban settings of Masaka and Kampala in South-Central Uganda. Low-cost sensors offer a more feasible option for continuous air quality monitoring in low-resource settings, but they often require location-specific evaluation and calibration. Most existing calibration models, however, have been designed for regions like the U.S. and China (Barkjohn et al., 2021; Giordano et al., 2021; Zheng et al., 2018). Although some localized calibration models have been developed, these have largely been developed using ambient PM_2.5_ measurements in urban locations and have not fully addressed the specific challenges of monitoring air quality in both urban and rural areas (Adong et al., 2022; Smith et al., 2023). To address this gap and enhance the accuracy of the low-cost sensor data, we developed a region-specific calibration model tailored for Uganda, considering both urban and rural environments. We then applied this calibration model to our network of low-cost air quality monitors to examine spatiotemporal trends in ambient PM_2.5_ concentrations in South-Central Uganda.

## 2. Materials and methods

### 2.1. Study domain

**Fig. 1.**
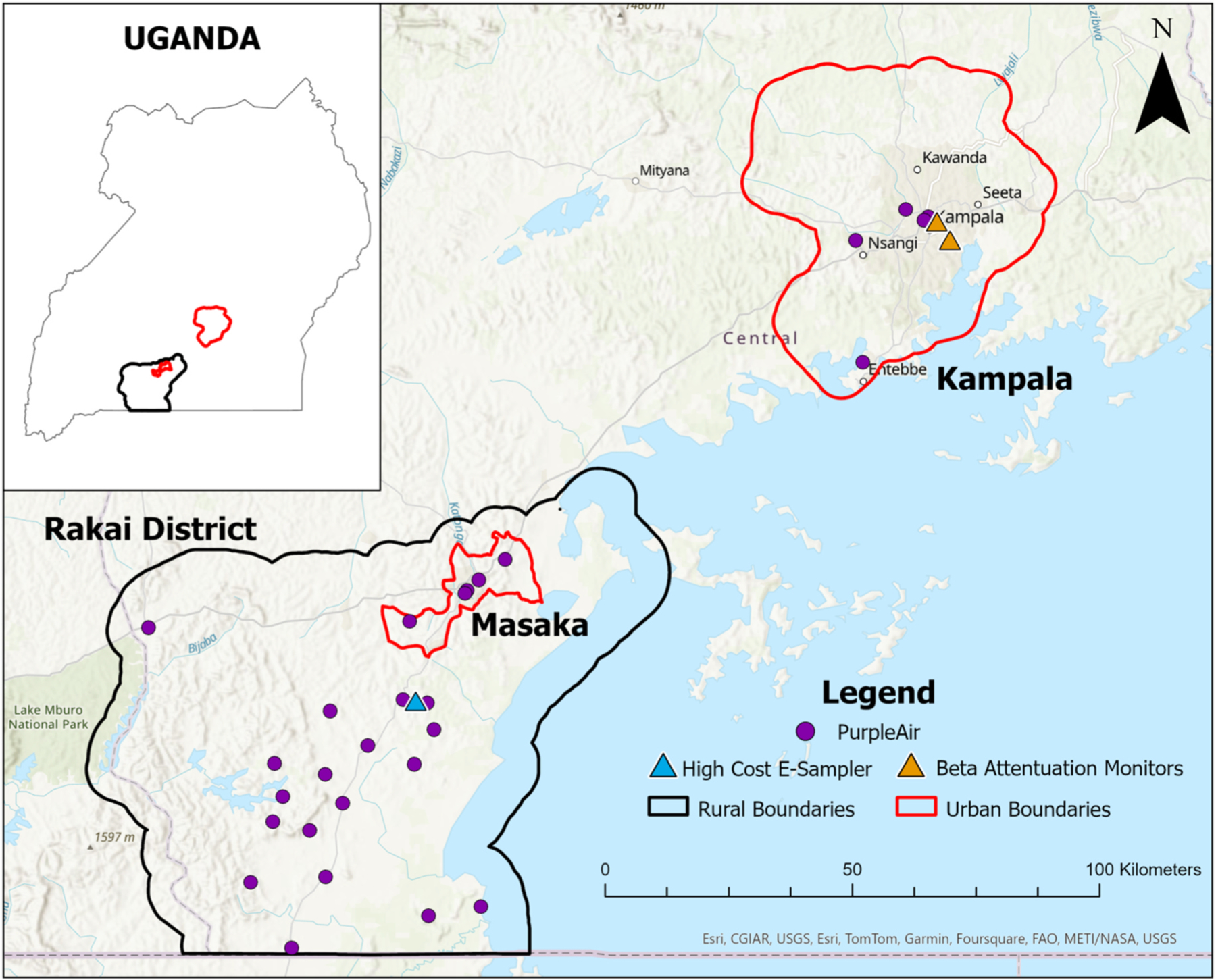
The distribution of air quality monitoring stations, including high-cost samplers/references and low-cost monitors within Kampala and the Rakai District of Uganda. The inset shows the location of the study domain within the national context. A network of 27 PurpleAir devices, shown in purple, were deployed throughout the Rakai District, Masaka and Kampala. The high-cost E-Sampler is represented by the blue triangle and the two Beta Attenuation Monitor are indicated in orange.

The study was conducted in the rural Rakai region and the urban centers of Masaka and Kampala, in South Central Uganda (Fig. 1). Uganda’s population was estimated to be approximately 45.9 million in 2024, with an annual growth rate of 2.9 % (Uganda Bureau of Statistics, 2024). Uganda is undergoing rapid urbanization, with urban populations increasing significantly in recent years (Tumwesigye et al., 2021). Kampala, the capital and largest city, grew at an average annual rate of 2.3 % from 2014 to 2024, and Masaka at 3.1 % during the same period (Uganda Bureau of Statistics, 2016; Tumwesigye et al., 2021). These rates are relatively high compared to the global urbanization rate, which averaged around 1.8 % annually from 1990 to 2018 (United Nations, 2019).

Located at the equator, Uganda’s climate is largely tropical, with mean annual temperatures across the country typically ranging from 16 °C to 30 °C throughout the year. The Northern and Eastern regions occasionally experience high temperatures exceeding 30 °C, whereas the South-Western region may have temperatures below 16 °C (Uganda Bureau of Statistics, 2016). The Central, Western, and Eastern regions historically follow a bi-modal rainfall pattern: the first rains occur between March and May, and the second between September and November. In 2022, the highest rainfall was recorded in September (1621 mm) and November (1534 mm), and the driest months were February (429 mm) and January (376 mm) (Uganda Bureau of Statistics, 2023). Monthly mean relative humidity was lowest in January at 73.8 % and highest in November at 86.3 % (Uganda Bureau of Statistics, 2023, 2023).

### 2.2. Ambient monitoring network

We established a monitoring network of low-cost sensors (PurpleAir PA-II-SD, PurpleAir, Draper, UT) across both urban (Masaka and Kampala) and rural (Rakai region) areas in South Central Uganda (Fig. 1). Device installation began in October 2022 and, while sampling is currently ongoing, this analysis includes data from October 27, 2022 to November 17, 2023. A total of 27 PurpleAir devices were installed, with 18 placed in the Rakai region, four in Masaka, and five in Kampala. Prior research has demonstrated strong inter-sensor agreement among PurpleAir devices under a variety of environmental conditions, supporting their application in ambient monitoring networks (Barkjohn et al., 2021; Kelly et al., 2017; Sayahi et al., 2019).

The PurpleAir sensors were installed at healthcare facilities supported by the Rakai Health Sciences Program (RHSP). Health facilities in Uganda are classified into several tiers: HC I (community outreach), HC II (parish level outpatient services), HC III (sub-county level with outpatient services, maternity, and inpatient wards), HC IV (county level with more comprehensive services, including surgery), and hospitals (Basajja and Nambobi, 2022). The sensors were installed at health facilities classified as tier 3 and above (HC III), which have access to internet and electricity, albeit intermittently. We placed the devices outside the facilities in locations facing open spaces to minimize potential airflow obstructions and reduce the influence of pollution from inside the building.

**Fig. 2.**
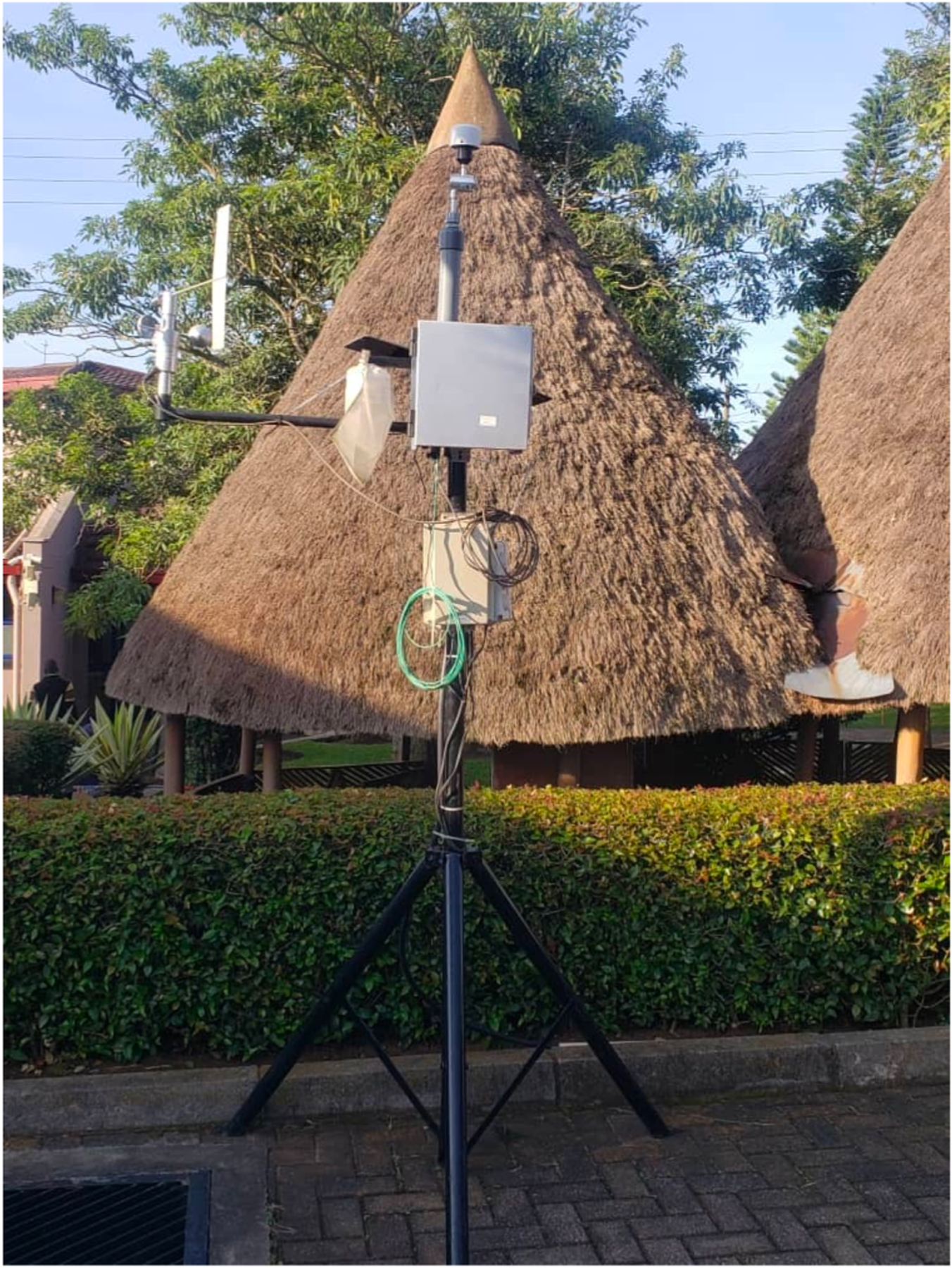
Colocated PurpleAir device placed inside a Nylon filter bag with the E-Sampler at Kalisizo, Uganda. The PurpleAir sensor was placed in a 74- µm pore size filter bag to protect the device from large dust particles and insects, ensuring unobstructed air flow and accurate measurements.

PurpleAir sensors (Plantower PMS5003), known for their cost-effectiveness and high temporal resolution (2-min), measure PM_2.5_ concentrations by detecting the light scattered by aerosols (Giordano et al., 2021; Levy Zamora et al., 2019; Wallace et al., 2021). The devices report mass concentrations of particles with aerodynamic diameters of <1 µm (PM_1_), <2.5 µm (PM_2.5_), and <10 µm (PM_10_); in this work we have focused on PM_2.5_. Ambient PM_2.5_ data was uploaded to the PurpleAir cloud real-time via Wi-Fi, and recorded directly onto an onboard SD card. To mitigate data loss during Wi-Fi outages, we manually downloaded data from the SD cards every three months and combined it with the real-time data from the online platform. To enhance the reliability of the sensors and reduce maintenance needs, each unit was placed inside a nylon filter bag (74-µm pore size, Fig. 2). The bags protect the sensors from large dust particles ≥74 µm and insects, which can obstruct the air inlet and interfere with the measurements. Considering that the pore size is significantly larger than the aerodynamic diameter of PM_2.5_ and that the low flow rate through the bag limits losses by impaction, we expect particle loss to be minimal. Side-by-side testing of sensors with and without the mesh bags showed no noticeable difference in PM_2.5_ measurements (data not shown), supporting their use as a practical way to minimize environmental contamination without affecting measurement accuracy.

To develop a calibration model for the PurpleAir data, we collocated a combined nephelometric and gravimetric sampler (E-Sampler, Met One Instruments, Inc. Grants Pass, OR USA) alongside the PurpleAir installed at the RHSP office in Kalisizo (Rakai region) in October 2022. The E-Sampler operates at a flow rate of 2.0 L per minute, passing the air through a laser optical module for real-time nephelometric readings at 5-min intervals. It includes an integrated dehumidifier to reduce the influence of relative humidity on nephelometric readings. In addition to nephelometric measurements, the E-Sampler captures particles on a 47-mm filter for gravimetric analysis. Although the E-Sampler has not been formally designated as a Federal Equivalent Method (FEM) by the U.S. Environmental Protection Agency (EPA), it is considered near-reference grade and has demonstrated compliance with FEM performance criteria in rigorous field conditions such as wildfire smoke (Holder et al., 2020; Mehadi et al., 2020).

Gravimetric samples were collected on Teflon filters with a 2 µm pore size for seven-day periods. Prior to each sample collection, we performed calibration of the E-Sampler’s ambient temperature, barometric pressure, relative humidity, and flow rate using a Kestrel weather meter (Kestrel Instruments, Boothwyn, PA, USA) and a TSI Mass Flowmeter 4100 (TSI Incorporated, Shoreview, MN, USA). Each calibration session included a leak test, and the E-Sampler performed an automatic 2.8-min self-test every hour during the sampling period, which checks for instrument functionality and data integrity. We used standardized data log sheets to record ambient temperature, barometric pressure, flow rate, and start and stop times for each one-week sampling interval.

#### 2.2.1. FEM monitors in Uganda

At the time of the study, two Beta Attenuation Monitors (BAMs, Met One Instruments Inc., Grants Pass, OR, USA) configured to measure PM_2.5_ were available in Uganda: one BAM1020 located at the U.S. Embassy, and another BAM1022 maintained by AirQo at Makerere University (Fig. 1). The two BAM monitors were approximately 4.5 km apart within Kampala city. The U.S. Embassy BAM was located near major roadways with heavy traffic and high human activity. In contrast, the AirQo BAM was located in a green space on Makerere University campus, away from major traffic. In March 2023, we collocated one PurpleAir device with the BAM at Makerere University. Data from the urban collocated site was limited to two weeks due to technical challenges, including intermittent electricity supply and Wi-Fi connectivity issues, which affected the PurpleAir’s ability to consistently record data. The short duration of data collection was insufficient for model calibration, so the PurpleAir sensor collocated with the BAM at Makerere University in Kampala was used solely for external validation of the calibration model’s performance in urban settings. Additionally, a significant data discrepancy was observed between the two BAMs, despite their close proximity. The U.S. Embassy BAM data consistently showed PM_2.5_ concentrations nearly 20 µg/m^3^ higher than those recorded by the AirQo BAM (Fig. S1). While differences in site conditions, such as nearby roads or burning activity, may have contributed to this difference, our initial analyses of the data suggested that a substantial bias in these measurements was probable. Thus, we chose not to include the U.S. Embassy BAM data in our further analyses, and used the AirQo BAM for evaluation of the model in Kampala.

### 2.3. Quality assurance/quality control (QA/QC) and data cleaning

#### 2.3.1 . PurpleAir QA/QC and data cleaning

Our data cleaning procedure for the PurpleAir PM_2.5_ measurements followed the methods outlined by Barkjohn et al. (2021, 2022). We used the PM data labeled [cf = 1] as it has been demonstrated to more strongly correlate with reference monitors compared to the [cf = atm] channel over a wide range of concentrations (Barkjohn et al., 2022). The 2-min PurpleAir data were aggregated to an hourly resolution. We then performed an hourly percent difference check between the two channels (sensors A and B) of each PurpleAir device. Data points were excluded if the deviation between sensors A and B exceeded 70 % and 5 µg/m^3^ (Barkjohn et al., 2022; Tryner et al., 2020). Implementing these criteria led to an exclusion of 1774 (1.1 %) out of 157,094 hourly data points across all sensors. Observations under 1 µg/m^3^ were replaced with 1/√2 µg/m^3^ to mitigate inaccuracies at very low concentration ranges (n = 570 observations, 0.4 % across all sensors).

#### 2.3.2. E-sampler QA/QC and data cleaning

Prior to field deployment, the 47-mm Teflon filters were conditioned for at least 24 h in a temperature and humidity-controlled laboratory at Johns Hopkins University, maintained at 21 °C ± 3 °C and 35 % ± 5 % humidity, respectively. The filters were then weighed following the procedures outlined by Fandiño-Del-Rio et al. (2020).

For quality assurance, we included three blank samples to control for background noise and potential contamination. To determine the Limit of Detection (LOD) for the gravimetric samples, we calculated three times the standard deviation of the blank samples, resulting in an LOD of 0.109 mg. Sample masses were blank corrected and any blank-corrected masses below the LOD were assigned a value equal to the LOD/√2 (0.0774 mg). This adjustment was applied to two (3.8 %) gravimetric samples. Nephelometric data with an hourly average under 1 µg/m^3^ were replaced with 1/√2 µg/m^3^ (n=570, 0.22 %). We then determined a weekly correction factor, defined as the ratio of the filter-based concentration to the weekly-averaged nephelometric concentration. Any correction factors below 1.5 times the interquartile range (IQR) lower than the first quartile (< 0.68) or above 1.5 times the IQR higher than the third quartile (> 2.10) were replaced by the median correction factor (1.30). This replacement was applied to 11 of the 51 correction factors (21.6 %). The weekly correction factor was applied to the 5-min nephelometric data during that seven-day period, and the gravimetrically corrected nephelometric data were aggregated to hourly averages to match the temporal resolution of the aggregated PurpleAir data. No additional calibration or correction was applied to the E-Sampler data beyond the gravimetric correction.

### 2.4. Calibration of PurpleAir monitors

We started our calibration model development with linear regression models, acknowledging their simplicity and widespread application in previous studies (Barkjohn et al., 2021). We also explored more complex approaches including machine learning algorithms such as Random Forest (RF) and XGBoost, along with Generalized Additive Models (GAM), and Autoregressive Integrated Moving Average with exogenous variables (ARIMAX) models. Covariates selected for our models included relative humidity (RH) in percent (%), temperature (T) in Celsius (°C), dew point (DP) in °C, and pressure (P) in hPa. These variables were chosen based on their documented influence on optical PM_2.5_ measurements in previous studies, which indicate that such factors can significantly affect sensor readings by altering particle light scattering and absorption (Barkjohn et al., 2021, 2022; Emekwuru and Ejohwomu, 2023; Giordano et al., 2021; Wallace et al., 2021). Temperature, RH, and pressure were measured by the PurpleAir sensor. Dew point temperature was calculated using the Magnus-Tetens formula, based on the ambient air temperature (in Celsius) and RH (Lawrence, 2005).

We used the hourly gravimetrically-corrected PM_2.5_ concentrations obtained with the E-Sampler (PM_2.5_) and the mean hourly PM_2.5_ concentrations computed from channels A and B of the collocted PurpleAir (PA_cf_1_) in Kalisizo from October 27, 2022, to November 17, 2023. We built a linear regression model of the hourly E-sampler PM_2.5_ concentrations as a function of all the independent variables and calculated residuals. We then excluded observations with residuals greater than two standard deviations, which resulted in the removal of 295 (3.92 %) data points. The final dataset included 7236 collocated PM_2.5_ hourly-averaged observations. The dataset was split into two portions: the first 80 % (5784 hours; 241 days) was used to train and evaluate the models, with the remaining 20 % (1452 hours) held out completely to test the models on unseen data (Hastie et al., 2009; Kuhn and Johnson, 2013a; Kuhn and Johnson, 2013b). The training set was used to develop the models, while the test set was used to assess their predictive accuracy. During model development, we applied time series cross-validation within the training set to ensure robust evaluation, using the caret package in R (Kuhn, 2008). The cross-validation started with an initial window of 576 hours (24 days) for training and the subsequent 576 hours as the validation subset. This process was repeated, each time increasing the training set by 576 hours and moving the validation set to the next contiguous subset of 576 hours. For hyperparameter tuning in our machine learning models, we set the tune length to 20.

For the ARIMAX model, we first imputed missing values in our time series with a random forest imputation method, using the misRanger package in R (Mayer, 2017). We then split our imputed data set into training (first 80 %) and test (remaining 20 %) datasets. We selected the order of the model through visual inspection of autocorrelation and partial autocorrelation plots, and trained the model on the imputed training dataset including the hourly PurpleAir PM_2.5_ concentrations and all the above-mentioned covariates as exogenous variables. We then used the model to forecast on the imputed test dataset, and evaluated its performance on the original test dataset.

Model performance was evaluated on the withheld test dataset using several key error metrics, including the root mean square error (RMSE), bias, mean absolute error (MAE), mean absolute percentage error (MAPE), and coefficient of determination (R^2^). To benchmark our model’s performance, we compared it with Barkjohn’s US-based correction model which is applied across the entire United States without distinction between urban and rural settings (Barkjohn et al., 2021). Since our goal was to develop a model applicable to both rural and urban regions in Uganda, this comparison provided a valuable reference point for evaluating the accuracy and broader applicability of our locally developed calibration model in a similar cross-environment context.

After selecting our final model, we applied the calibration to all the PurpleAir monitors in our study. Any corrected concentrations < 1 µg/m^3^ were replaced by 1/√2 µg/m^3^ to mitigate inaccuracies at low concentrations. To assess the model’s applicability to urban sites with PurpleAir sensors, we compared the corrected concentrations of the PurpleAir device collocated with the AirQo BAM measurements in Kampala. Although the AirQo BAM data were not used in model development, they served as an external reference to evaluate how well our calibration model performed in an urban setting. Lastly, we used the calibrated PurpleAir data to examine the spatiotemporal trends in ambient PM_2.5_ concentrations in South-Central Uganda.

## 3. Results

### 3.1. Calibration model

We summarized performance metrics in Table 1 for various calibration models applied to the PurpleAir PM_2.5_ measurements. Uncorrected ambient PM_2.5_ measurements from the collocated PurpleAir sensor overestimated those of the E-Sampler, with a bias of 15.96 µg/m^3^, an RMSE of 20.37 µg/m^3^, MAE of 15.97 µg/m^3^, and MAPE of 134.62 %, highlighting the need for calibration to improve accuracy. In comparison, Barkjohn et al. (2021) reported a lower RMSE of 8 µg/m^3^ for raw PurpleAir data in the U.S., indicating that the overestimation in our study may be more pronounced in the context of Uganda’s ambient air quality.

Model 1, which only included PurpleAir PM_2.5_ measurements as the predictor, showed a substantial reduction in errors and bias compared to the uncorrected PurpleAir measurements, with an RMSE of 3.95 µg/m^3^, MAE of 2.71 µg/m^3^, MAPE of 25.15 %, and a minimal bias of −0.270 µg/m^3^ suggesting a slight underestimation in PM_2.5_ concentrations. Among the other basic linear models (Models 2–5), Model 3 which included humidity, displayed the lowest RMSE (3.83 µg/m^3^) and MAE (2.62 µg/m^3^). Adding interactions of humidity with temperature (Model 6) or dew point (Model 9) did not improve model accuracy. Model 10, which added an interaction term between humidity and pressure, had the lowest MAE (2.60 µg/m^3^) among all the linear models tested, but a similar RMSE (3.83 µg/m^3^) and larger bias (−0.404 µg/m^3^) compared to Model 3. All linear models (Models 1–11) demonstrated high predictive power with R^2^ values of 0.91–0.92.

Surprisingly, the machine learning models we tested did not outperform the regression-based models. While both the Random Forest model (Model 12) and tuned XGBoost model (Model 13) had a lower bias than all other models, they had a higher variance in errors compared to the best-performing linear model (Table 1). Time series models, particularly the GAM (Model 15) and the ARIMAX (Model 14), were evaluated for their ability to handle temporal patterns. The GAM performed well with the lowest RMSE (3.81 µg/m^3^) among all models tested, a bias of −0.294 µg/m^3^, and MAE of 2.64 µg/m^3^. The ARIMAX model on the other hand, had higher errors than the best-performing linear model, with an RMSE of 3.95 µg/m^3^, MAE of 2.74 µg/m^3^, and MAPE of 30.62 %, and the largest bias (−1.047 µg/m^3^) among all models tested.

After evaluating the performance metrics of all our models, we selected Model 3 (Eq. (1)) as the final calibration model due to its balance of accuracy and complexity:

Eq. 1 $$PA_{cal}=2.9271+0.4994\times PA_{cf\_1}-0.0675\times RH$$

where PA_cal_ denotes the calibrated PM_2.5_ concentration in µg/m^3^, PA_cf_1_ corresponds to the mean PurpleAir cf_1 data computed from channels A and B (µg/m^3^), and RH is the relative humidity (%). The model’s robustness was confirmed by a residual standard error of 4.44 µg/m^3^ on 5781 degrees of freedom, a multiple R^2^ of 0.93, and a similarly high adjusted R^2^, underscoring the model’s effectiveness in prediction.

In Fig. S2, we summarize the performance of three calibration models—Linear (Model 3), Random Forest (Model 12), and XGBoost (Model 13) —against the E-Sampler PM_2.5_ measurements. The tight clustering of points around the linear fit lines suggests that each model effectively captures the linear relationship between predicted and actual values. All three models showed high Spearman’s rho values, indicating strong performance in capturing the variability of PM_2.5_ concentrations. In Fig. S3, we present a Bland-Altman plot that examines the agreement between the corrected PurpleAir data using the final calibration model (Eq. (1)) and the E-Sampler measurements. The solid horizontal line represents the mean difference (bias) between the two measurement methods, which is approximately −0.07 µg/m^3^. A near-zero mean difference indicates negligible bias in the linear model’s predictions compared to the E-Sampler, suggesting good overall agreement. The plot shows the limits of agreement at 8.40 µg/m^3^ and −8.55 µg/m^3^, respectively. Most data points lie between these limits, confirming that the discrepancies between the two sets of measurements are acceptable across most observations. However, a slight trend was observed: as the average PM_2.5_ concentration increases, the difference between the corrected PurpleAir and E-Sampler measurements tends to become more negative, suggesting an increasing negative bias at higher concentration levels.

**Fig. 3.**
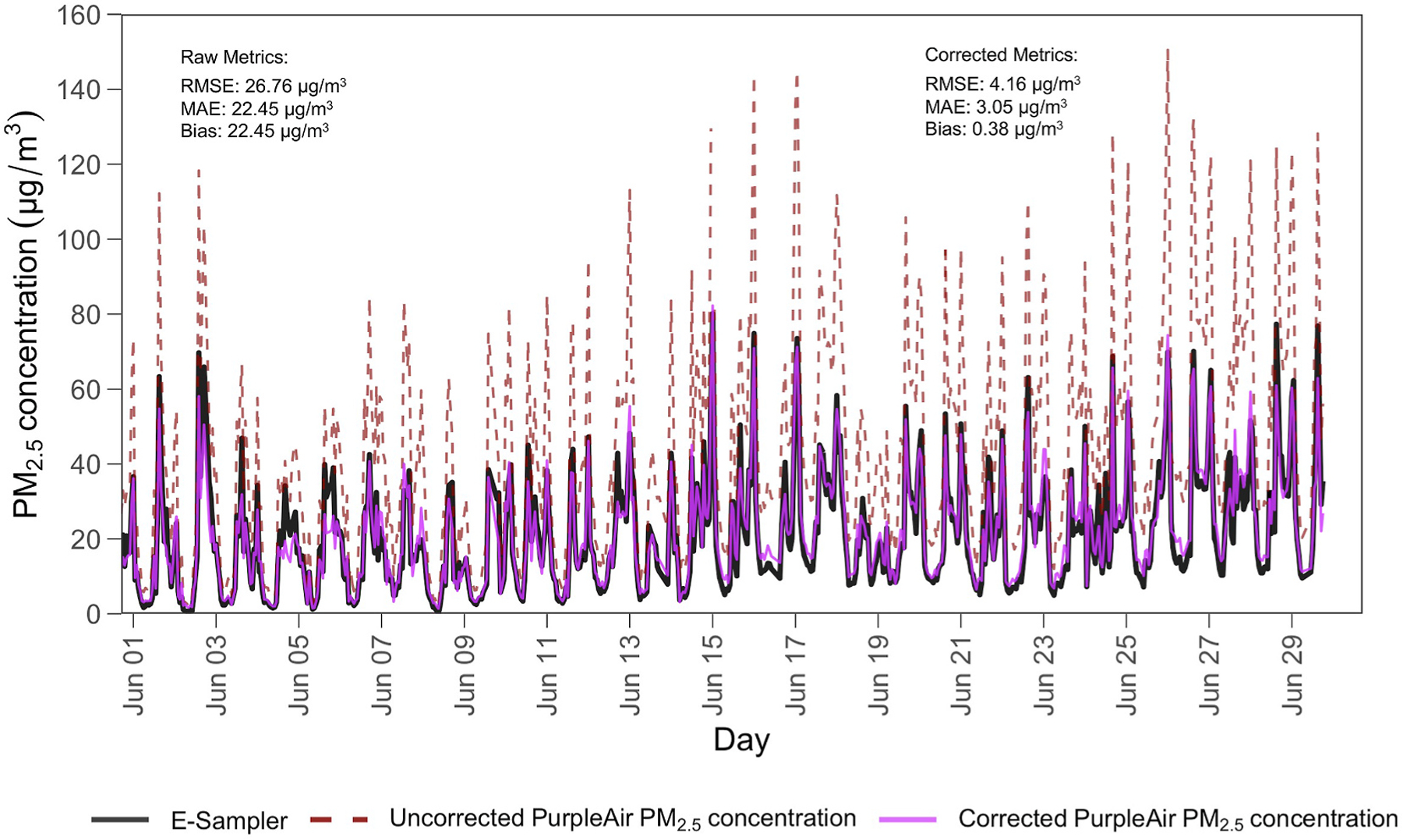
Hourly-averaged PM_2.5_ concentrations in July 2023, at the collocation site in Kalisizo. The dashed red line depicts the raw PurpleAir PM_2.5_ concentration, solid black the high-cost E-Sampler and the solid purple line represents the corrected PurpleAir concentration.

In Fig. 3, we display the daily ambient PM_2.5_ concentration data for June 2023, comparing raw and corrected outputs from the collocated PurpleAir sensor against measurements from the E-Sampler at Kalisizo. Significant disparities between the raw PurpleAir data and the E-Sampler data are illustrated, with the raw PurpleAir data generally overestimating ambient PM_2.5_ concentrations compared to the E-Sampler. Raw data from the PurpleAir had an RMSE of 26.76 µg/m^3^, MAE of 22.45 µg/m^3^, and bias of 22.45 µg/m^3^ highlighting poor initial agreement with the E-Sampler data. After applying the calibration model, the PurpleAir data aligned much closer to the E-Sampler readings, with the RMSE reducing to 4.16 µg/m^3^ , MAE to 3.05 µg/m^3^, and bias to 0.38 µg/m^3^. Overall, our local calibration model showed significantly improved performance compared to the raw data, making it more reliable for accurate PM_2.5_ estimation in this regional setting.

A direct comparison between our local calibration model and Barkjohn’s U.S.-based calibration model (Barkjohn et al., 2021) was conducted to gauge its effectiveness in a different regional setting. The comparison revealed that our model consistently outperformed the U. S.-based model across several key metrics (Table 1). Our local model demonstrated a tighter fit to the observed data, with an RMSE of 3.83 µg/m^3^ compared to 4.34 µg/m^3^ for the U.S.-derived model. Furthermore, the bias in our model was significantly lower at −0.387 µg/m^3^, indicating minimal systematic deviation, whereas the U.S.-derived model had a bias of 2.21 µg/m^3^, suggesting a tendency towards overestimation. Our local model also had lower MAE (2.62 µg/m^3^ vs 3.51 µg/m^3^) and MAPE (30.97 % vs 65.85 %) values than the U.S.-derived model, pointing to smaller average errors and percentage errors in our predictions.

To evaluate the applicability of our calibration model developed from rural data in urban settings, we applied it to PurpleAir data at the collocation site at Makerere University in Kampala and assessed its performance against the AirQo BAM data. As shown in Fig. S5, the corrected PurpleAir measurements closely align with the AirQo BAM data at that site, with a slight underestimation of some peaks. The raw metrics for the uncorrected PurpleAir data show an RMSE of 18.37 µg/m^3^, an MAE of 11.32 µg/m^3^ and a bias of 9.23 µg/m^3^ compared to the reference BAM data. After applying the calibration model, the RMSE improved to 10.10 µg/m^3^, the MAE to 6.85 µg/m^3^, and the bias to −5.29 µg/m^3^. These results demonstrate that the calibration model performs well in urban settings, enhancing the overall accuracy of PM_2.5_ estimates.

### 3.2. Spatial and temporal trends

Seasonal hourly ambient PM_2.5_ concentrations, as summarized in Table 2, revealed statistically significant variations between dry and wet seasons, and across rural and urban regions. Mann-Whitney U tests were conducted to compare PM_2.5_ concentrations within each region across seasons and between rural and urban regions for each season. In rural areas, the mean (SD) PM_2.5_ concentration during the dry season was 22.1 (16.2) µg/m^3^ which decreased to 12.7 (12.2) µg/m^3^ during the wet season (p < 0.001). In urban areas, mean PM_2.5_ concentrations were 71 % and 83 % higher than in rural regions during the dry and wet seasons, respectively. The mean dry season concentration in urban areas was 37.7 (30.0) µg/m^3^, and the wet season concentration was 23.2 (22.2) µg/m^3^ (p < 0.001 for both seasonal and regional comparisons).

**Fig. 4.**
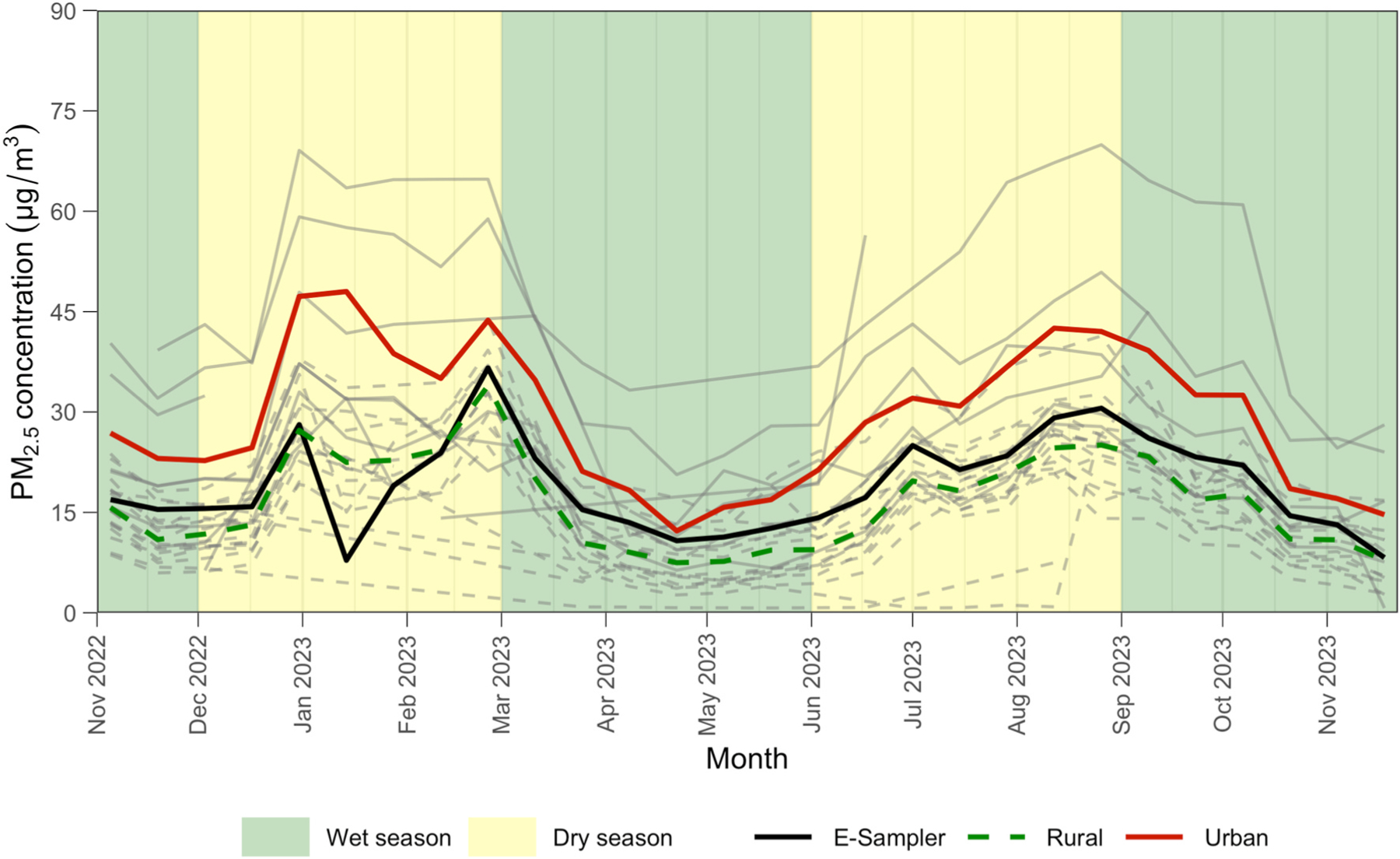
Biweekly PM_2.5_ Concentrations Across Rural and Urban Regions from October 2022 to November 2023. Mean corrected PM_2.5_ concentrations across all rural and all urban locations are shown in the dashed green and solid red line, respectively. The E-Sampler data from Kalisizo is depicted with a solid black line. The grey lines in the background represent measurements from individual sites across our network (solid lines for urban and dashed lines for rural). The wet and dry seasons are denoted by the green and yellow shaded areas, respectively.

**Fig. 5.**
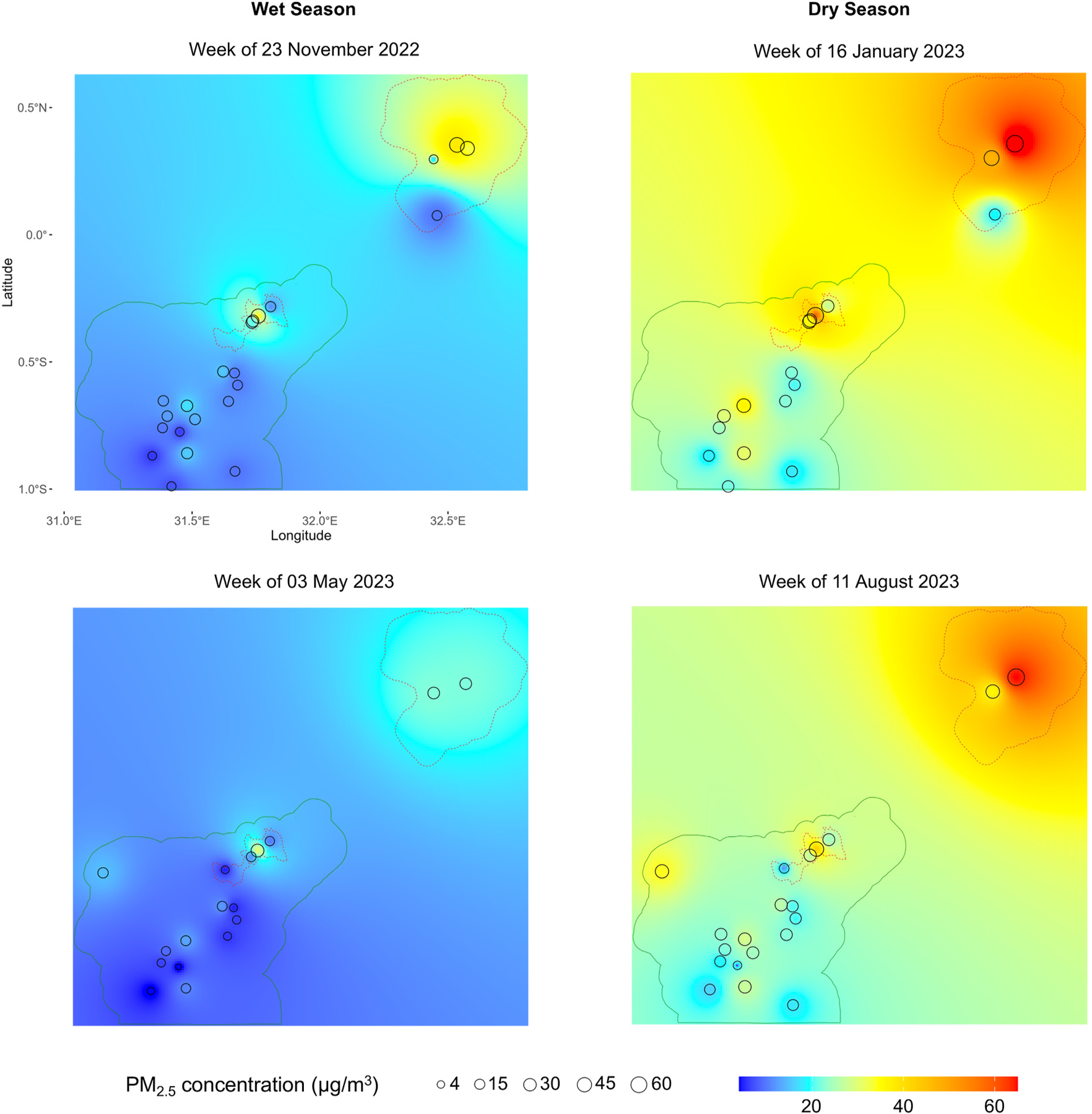
Spatial interpolation maps of PM_2.5_ concentrations during the wet and dry seasons. Weekly averages are shown in November 2022, January 2023, May 2023 and August 2023 to demonstrate variability. Plots on the left denote the wet season and plots on the right represent the dry season. The red boundaries represent the urban regions of Kampala and Masaka whereas the green boundary represents the rural Rakai district. The varying sizes of the circles represents the corrected average weekly concentration of PM_2.5_ at each PurpleAir site.

In Fig. 4 we display biweekly-averaged PM_2.5_ concentrations from October 2022 to October 2023, comparing measurements between urban and rural sites. Urban PM_2.5_ concentrations are consistently higher than those in rural areas, with observed peaks during the dry season in February 2023 and August 2023. To examine ambient PM_2.5_ concentrations throughout our entire study domain, we also created spatial interpolation maps on selected weeks during both wet and dry seasons using inverse distance weighting (Fig. 5). Consistent with the results shown in Fig. 4, PM_2.5_ concentrations during the wet season are generally lower across the study domain compared to the dry season, and urban areas, particularly Kampala, consistently exhibit higher PM_2.5_ concentrations compared to rural areas. We also observe a larger variability in ambient PM_2.5_ concentrations across urban sites compared to rural sites (Fig. 4), which suggests more localized sources of air pollution, such as vehicle emissions and industry, in urban settings.

**Fig. 6.**
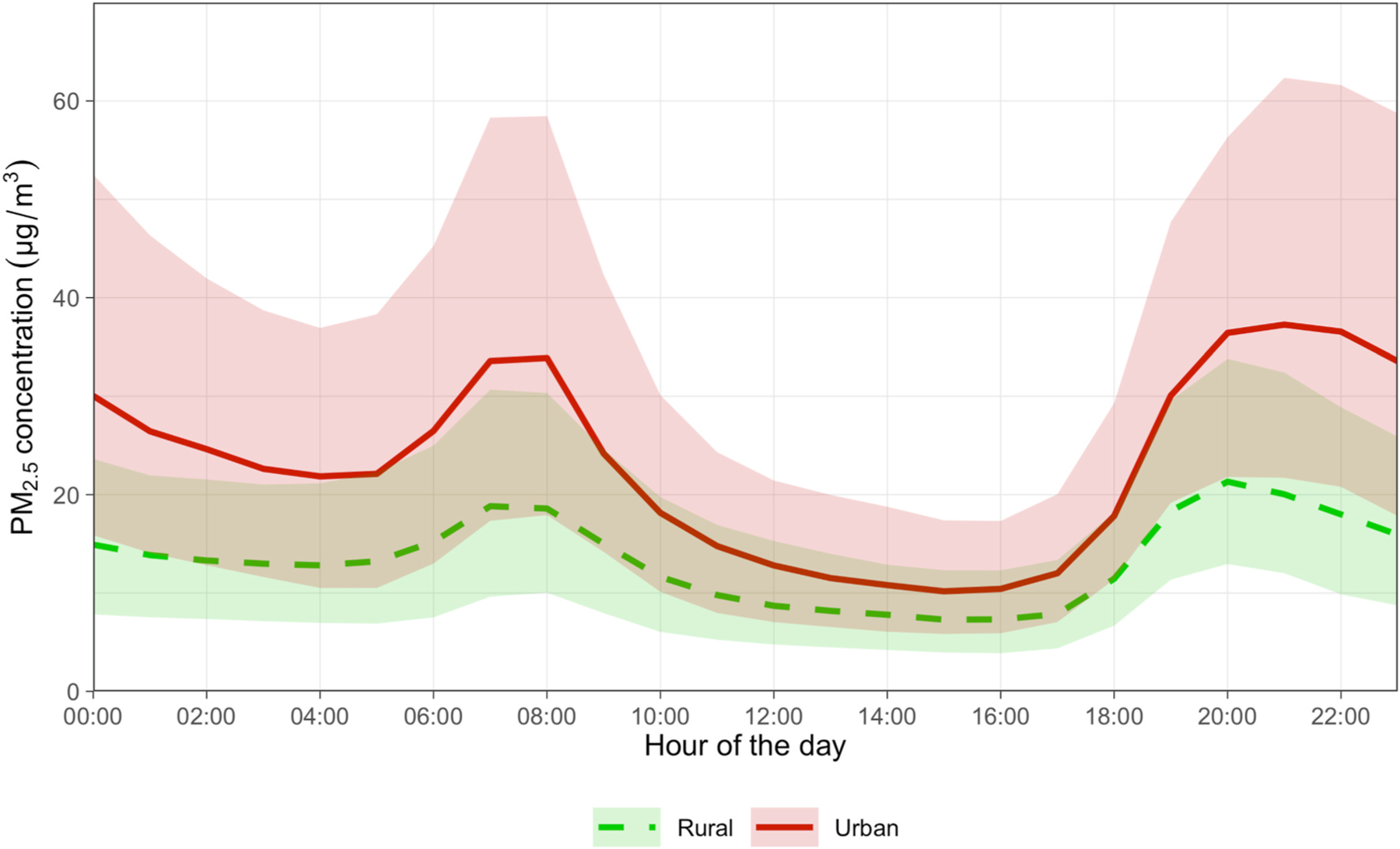
Diurnal variations in PM_2.5_ concentrations from October 2022 to November 2023, comparing rural and urban settings. Each time series illustrates the median hourly PM_2.5_ concentrations throughout the day in rural (green dashed) and urban (red solid) environments. The shaded areas indicate hourly PM_2.5_ concentrations between the 25th and 75th percentile for each hour.

Diurnal patterns of ambient PM_2.5_ concentrations are similar across urban and rural locations (Fig. 6). Both urban and rural settings display a cyclical pattern, with a peak in PM_2.5_ concentrations in the morning, from approximately 6 a.m. to 9 a.m., and a second, slightly higher peak in the late evening around 6 p.m. to 10 p.m.

## 4. Discussion

This study examines ambient PM_2.5_ concentrations across urban (Masaka, Kampala) and rural (Rakai region) settings in South Central Uganda. Using a network of 27 low-cost PurpleAir sensors, we established the largest monitoring network in the region outside of the extensive AirQo network which is predominantly spread across Kampala only. The deployment, which began in October 2022, significantly expanded the air quality monitoring network in the country, particularly in rural areas previously lacking data. A key aspect of our study was the development and application of a region-specific calibration model to improve the accuracy of the low-cost sensor data.

We explored multiple models to improve measurement accuracy; however, the simplicity and interpretability of the linear model are helpful for practical applications in resource-limited settings. The more complex GAM model offered slight improvements in RMSE and bias but did not significantly outperform the linear model, while the XGBoost and RF models offered improvements in bias but did not outperform the linear model in terms of RMSE. The locally tailored calibration model outperformed an existing US-derived model (Barkjohn et al., 2021), with lower bias and errors in both absolute and percentage terms when applied to our study domains. These results indicate that our model provided more accurate estimates of local PM_2.5_ concentrations than the US-based model. The superior performance of our region-specific model can be attributed to the sensitivity of PurpleAir PM_2.5_ measurements to local environmental and pollution characteristics.

Our region-specific calibrated data show that there are distinct seasonal variations in ambient PM_2.5_ concentrations, with higher concentrations during the dry season than the wet season as previously observed (Guttikunda and Gurjar, 2012; Jain et al., 2020; Tefera et al., 2020). However, it is important to note that the traditional definitions of wet and dry seasons in Uganda are becoming less predictable due to climate change. The wet seasons are often shorter or arrive later than expected, which could affect the interpretation of our findings (Intergovernmental Panel On Climate Change (Ipcc), 2023). Our results also highlight the significant impact of urbanization on air quality, with urban areas showing consistently higher pollution levels than rural areas. Similar studies have observed the influence of urbanization on air quality in different regions, including Chinese cities, various countries, and locations in Africa and Southern California (Han et al., 2014; Li et al., 2019; Okure et al., 2022; Ren et al., 2022; Wang et al., 2020). During the dry season, urban areas in our study recorded mean ambient PM_2.5_ concentrations of 37.7 µg/m^3^, compared to a mean of 23.2 µg/m^3^ in the wet season. We observed higher PM_2.5_ concentrations in Kampala compared to Masaka, with means of 47.0 µg/m^3^ vs 32.5 µg/m^3^ in the dry season, and 28.4 µg/m^3^ vs 20.5 µg/m^3^ in the wet season, respectively. Although these concentrations are lower than Kampala’s previously reported average of 55.7 ± 20.3 µg/m^3^, they are consistent with concentrations observed in other urban centers across SSA where pollution levels typically exceed WHO guidelines (Awokola et al., 2022; World Health Organization, 2021 ). In a 2014 study in the Mpererwe district of Kampala, high ambient PM concentrations were recorded with 24-h average concentrations of both fine and coarse particulate matter consistently above 100 µg/m^3^ during two sampling periods between December 2012 and January 2013 (Schwander et al., 2014). Additionally, a study in Dar es Salaam, Tanzania reported 24-h average urban PM_2.5_ levels of 30.1 µg/m^3^, which increased to 48.8 µg/m^3^ at high-traffic sites during a monitoring period spanning six consecutive weeks in the dry season (Njee et al., 2016).

In rural settings, mean ambient PM_2.5_ concentrations were 22.1 µg/m^3^ during the dry season and 12.7 µg/m^3^ in the wet season. These values fall within the range of ambient PM_2.5_ concentrations observed in other rural settings in East Africa. A study conducted at a rural site near Nairobi, Kenya, reported a mean daytime PM_2.5_ concentration of 10.7 ± 3.8 µg/m^3^ during a two-week sampling period in July 2009, which typically aligns with the dry season in the region (Kinney et al., 2011). Research in rural Ethiopia has documented an average annual PM_2.5_ concentration of 27.9 µg/m^3^, attributed to the high consumption of agricultural residues—such as animal dung and crop stalks and stubbles—as cooking fuel (Benti et al., 2021). The PM_2.5_ concentrations in the rural region could be attributed to a combination of agricultural activities, natural dust emissions, and possibly household biomass burning, which are significant contributors to air pollution in these settings (Mulenga et al., 2018; Tessema et al., 2022).

Our study in South Central Uganda has several strengths. First, we significantly expanded air quality monitoring in rural areas through the strategic deployment of low-cost PurpleAir sensors. This extensive network provides enhanced spatial coverage and contributes valuable insights into localized and regional air quality trends. Another major strength of our study is the development and implementation of a locally tailored calibration model, akin to Barkjohn’s U.S.-wide model but adapted for the specific atmospheric and pollution conditions of Uganda. Additionally, our analysis of seasonal variations in PM_2.5_ concentrations across urban and rural settings offers detailed insights into the temporal dynamics of air quality in Uganda. The higher PM_2.5_ concentrations during the dry season suggest the impact of increased dust due to lower precipitation, while consistently higher ambient PM_2.5_ concentrations in urban compared to rural areas point to emissions from traffic and industry. In contrast, PM_2.5_ levels in the rural regions are likely driven by the widespread use of biomass fuels for cooking and heating. The observed PM_2.5_ peaks from 6 a.m. to 9 a.m. and 6 p.m. to 10 p.m. (Fig. 5) coincide with peak traffic hours, and could reflect increased vehicular emissions during these times (Hamza et al., 2023; Booysen et al., 2022).

Our study also has some potential shortcomings. We found that it was challenging to acquire high-quality ambient PM_2.5_ data in the urban setting. To make best use of the limited urban data available, we used the PurpleAir sensor collocated with the AirQo BAM in Kampala to externally validate the calibration model and assess its performance in an urban environment. This allowed us to verify our model’s performance against external standards, which is crucial for ensuring its applicability and robustness in diverse settings. Obtaining comprehensive data in rural settings in our study was only possible because of our active measurements, highlighting the difficulty in obtaining such data without dedicated efforts. Second, we relied on one E-Sampler in rural areas to calibrate our entire sensor network and one BAM in an urban area for external validation of that calibration. Additional regulatory-grade instruments could improve the generalizability of our calibrated measurements. Third, high solar radiation in tropical environments can elevate internal temperatures within sensor enclosures, especially in outdoor settings with prolonged sun exposure. As such, we include the sensor internal temperature and relative humidity in our calibration. However, it is possible that this calibration cannot fully account for the evaporation of semi-volatile organic compounds, potentially still influencing the calibrated PM_2.5_ measurements (Regmi et al., 2022). Lastly, we did not conduct chemical analysis or source apportionment of PM_2.5_ in this study. The lack of information on particle composition limits our ability to interpret potential differences in light scattering responses between instruments, as composition can influence optical measurements.

Despite these constraints, our study demonstrates the utility of low-cost sensor networks in regions traditionally underserved by air quality monitoring infrastructure. Looking forward, it is essential for future efforts in Uganda to achieve more equitable air quality data collection by expanding monitoring networks in both urban and rural areas. Establishing more sensors and collecting a comprehensive set of data will provide a clearer understanding of air quality across different settings, enabling more accurate assessments of health impacts and more tailored policy interventions. In urban areas, these findings can inform decisions around traffic management and land use planning, by identifying hotspots near roads, markets, schools, and industrial areas. High-resolution air quality data can support the implementation of traffic flow improvements or public transport infrastructure in areas with elevated PM_2.5_ concentrations. Detailed spatial and temporal data can also guide where to prioritize zoning changes to reduce ambient concentrations. In rural areas, expanded monitoring can help characterize ambient PM_2.5_ concentrations in communities that rely heavily on biomass or charcoal for cooking and areas where agricultural burning is common. Although ambient data cannot directly isolate the contribution of household sources, it can reveal whether areas with widespread residential combustion have persistently high ambient air pollution concentrations, which could help prioritize regions for targeted exposure studies. The monitoring network can also support comparisons across different times of the year to assess the impact of agricultural burning seasons relative to baseline conditions. These insights can inform regionally tailored interventions, such as cleaner cooking programs, agricultural burn management strategies, or community-level education campaigns to reduce exposure.

Improving monitoring coverage across both urban and rural areas can provide the evidence needed to inform targeted policy responses and reduce the current data disparity, ensuring that air pollution’s health impacts are recognized and addressed more equitably across Uganda.

## 5. Conclusion

We characterized spatiotemporal dynamics of ambient PM_2.5_ concentrations in South-Central Uganda and demonstrated the role of low-cost sensor networks in enhancing air quality research and surveillance in regions lacking air pollution data. By utilizing these technologies, we filled a significant gap in the environmental monitoring landscape, providing critical insights into areas where previous data scarcity hindered effective air quality management. Our work illustrates the utility of tailored calibration models based on local meteorology to improve the accuracy of low-cost air quality measurements. Our approach can be adapted for similar studies in other settings. Our findings on the distinct urban-rural disparities and seasonal variations in PM_2.5_ concentrations highlight the necessity for air quality management strategies that address the diverse conditions of each area. This study lays the groundwork for continued research on air quality in Uganda and supports public health efforts to reduce the health impacts of air pollution through precise interventions and informed policymaking.

## Supplementary Material

Supplement

## Figures and Tables

**Table 1:** Summary of the performance metrics for various correction models applied to the E-Sampler PM_2.5_ data. The models range from simple linear regressions to more complex Random Forest and XGBoost machine learning algorithms, as well as ARIMA and GAM time series models. Each model is described by its functional form (Equation), and the evaluation of each model performance is quantified using metrics including Root Mean Square Error (RMSE), bias, Mean Absolute Error (MAE), Mean Absolute Percentage Error (MAPE), and the coefficient of determination (R^2^). All metrics were calculated using predictions on a withheld 20 % test set. The selected final model is marked with an asterisk.

	Model	Equation	RMSE (µg/m^3^)	Bias (µg/m^3^)	MAE (µg/m^3^)	MAPE (%)	R^2 a^
*Raw Concentrations Linear*			20.37	15.96	15.97	134.62	0.92
	**1**	PM_2.5_ = β_0_ + β_1_PA_cf_1_	3.95	−0.270	2.71	25.15	0.92
	**2**	PM_2.5_ = β_0_ + β_1_PA_cf_1_ + β_2_T	3.84	−0.235	2.65	32.36	0.92
	3*	PM_2.5_ = β_0_ + β_1_PA_cf_1_ + β_2_RH	3.83	−0.387	2.62	30.97	0.92
	**4**	PM_2.5_ = β_0_ + β_1_PA_cf_1_ + β_2_DP	3.95	−0.196	2.73	25.99	0.92
	**5**	PM_2.5_ = β_0_ + β_1_PA_cf_1_ + β_2_P	3.95	−0.282	2.71	25.07	0.92
	**6**	PM_2.5_ = β_0_ + β_1_PA_cf_1_ + β_2_T + β_3_RH + β_4_T×RH	3.84	−0.214	2.74	34.37	0.92
	**7**	PM_2.5_ = β_0_ + β_1_PA_cf_1_ + β_2_T + β_3_DP + β_4_T×DP	3.85	−0.173	2.67	32.49	0.92
	**8**	PM_2.5_ = β_0_ + β_1_PA_cf_1_ + β_2_T + β_3_P + β_4_T×P	3.84	−0.247	2.64	31.32	0.92
	**9**	PM_2.5_ = β_0_ + β_1_PA_cf_1_ + β_2_RH + β_3_DP + β_4_RH×DP	3.84	−0.189	2.69	34.06	0.92
	**10**	PM_2.5_ = β_0_ + β_1_PA_cf_1_ + β_2_RH + β_3_P + β_4_RH×P	3.83	−0.404	2.60	29.41	0.92
	**11**	PM_2.5_ = β_0_ + β_1_PA_cf_1_ + β_2_DP + β_3_P + β_4_DP×P	3.97	−0.187	2.76	28.18	0.91
*Machine Learning*	**12**	Random Forest (Tuned) – [ntree = 100, mtry = 4]	3.87	−0.035	2.71	27.82	0.92
	**13**	XGBoost (Tuned) – [nrounds = 100, eta = 0.1, max depth = 4]	3.84	−0.086	2.66	30.43	0.92
*Time Series*	**14**	Autoregressive Integrated Moving Average Model with exogenous variables	3.95	−1.047	2.74	30.62	0.92
		ARIMAX(24,1,6) with PA_cf_1_, RH, T, P and DP					
	**15**	Generalized Additive Model (GAM)	3.81	−0.294	2.64	30.28	0.92
		PM_2.5_ = β_0_ + s(PA_cf_1_) + s(RH)					
*Barkjohn’s U.S. Correction*		PM_2.5_ = 5.75 + 0.524×PA_cf_1_ – 0.0862×RH	4.34	2.21	3.51	65.85	0.92

a R^2^ is computed from Pearson’s Correlation Coefficient.

**Table 2:** Summary statistics of average hourly seasonal PM_2.5_ concentrations in rural and urban regions from October 2022 to October 2023. The table presents total observation counts, mean seasonal PM_2.5_ concentrations, standard deviations, medians and interquartile ranges, stratified by rural and urban regions across dry and wet seasons, and for Masaka and Kampala separately. Mann-Whitney U tests were performed to compare seasonal differences within rural and urban areas, as well as differences between rural and urban areas for each season. An interaction test using a non-parametric factorial design was conducted to examine the combined effects of location and season on PM_2.5_ concentrations. Significant differences (p < 0.001) between rural and urban areas across seasons are marked with ***.

Region	Monitors (n)	Season	Observations (n)	PM_2.5_ concentration (µg/m^3^)		Median	Interquartile range
				Mean	Standard deviation		
**Rural**	18	Dry	57,375	22.08***	16.16	18.35	11.12 – 28.38
		Wet	55,488	12.70***	12.21	9.39	4.85 – 16.92
**Urban (Masaka** + **Kampala)**	9	Dry	21,162	37.69***	29.96	28.71	16.33 – 49.98
		Wet	18,730	23.22***	22.22	16.31	7.91 – 31.20

**Masaka**	4	Dry	13,730	32.52	26.19	24.47	14.49 – 42.70
		Wet	11,706	20.53	18.88	14.61	7.46 – 27.25
**Kampala**	5	Dry	7,432	47.01	33.82	38.31	21.82 – 62.91
		Wet	7,024	28.38	26.82	20.82	9.35 – 38.23

*** p < 0.001.
