# Supplementary material for: Calibration of low-cost sensor data for ambient PM2.5 monitoring across urban and rural settings in South Central Uganda
Source: Atmos Pollut Res. Author manuscript; Available in PMC 2025 Dec 3. (PMC12671985; doi:10.1016/j.apr.2025.102580)

SUPPLEMENTARY MATERIAL

Calibration of Low-Cost Sensor Data for Ambient $PM_{2.5}$ Monitoring Across Urban and Rural Settings in South Central Uganda

Sophia Le, William Checkley, Lauren Dudley, Joseph Ssuuna, Anthony Ndyanabo, Engineer Bainomugisha, Joel Ssematimba, Richard Sserunjogi, Deo Okedi, Deo Okure, Joseph Kagaayi, Larry Chang, Kirsten Koehler, Laura Nicolaou

Table of Contents

FIGURE S1 2
Time series comparison of hourly $PM_{2.5}$ concentrations measured by AirQo BAM and U.S. Embassy BAM devices from April 1st to April 18th, 2023. The x-axis represents the date, while the y-axis shows the $PM_{2.5}$ concentrations in micrograms per cubic meter (µg/m³). The green line represents the AirQo BAM measurements, and the black line represents the U.S. Embassy BAM measurements.

FIGURE S2 3
Scatter plot comparing the performance of three correction models against the E-Sampler hourly-averaged $PM_{2.5}$ concentration measurements. The x-axis represents the E-Sampler $PM_{2.5}$ concentrations in micrograms per cubic meter (µg/m³). The y-axis corresponds to the corrected PurpleAir $PM_{2.5}$ concentrations predicted by the Linear (Model 3), Random Forest, and XGBoost models. On each panel the red line represents the line of best fit. Spearman's rho quantifies the strength of the correlation between observed and predicted values.

FIGURE S3 4
Bland-Altman plot of the agreement between the collocated corrected PurpleAir and E-Sampler hourly-averaged $PM_{2.5}$ concentration measurements at Kalisizo, Uganda. The x-axis displays the mean of the corrected PurpleAir and E-Sampler $PM_{2.5}$ concentrations). The y-axis shows the difference between the corrected PurpleAir and E-Sampler $PM_{2.5}$ concentrations. The solid horizontal line represents the mean difference (-0.07 µg/m³), and two dashed horizontal lines indicate the upper and lower limits of agreement (8.40 µg/m³ and -8.55 µg/m³, respectively). The blue line shows the trend in the bias across the range of measurements. The negative slope of the blue line suggests that the difference between the two methods increases as $PM_{2.5}$ concentration increases. This negative difference indicates that the corrected PurpleAir measurements are generally lower than the E-Sampler measurements at higher concentrations.

FIGURE S4 5
Comparative Analysis of $PM_{2.5}$ Concentrations Using Different Linear Calibration Models. This figure presents a comparison between corrected PurpleAir $PM_{2.5}$ concentrations using various models and the near reference grade E-Sampler measurements. Each plot aligns E-Sampler measurements (x-axis) against model predictions (y-axis) for Raw Data (note the different scale for the left panel), Model 1, Model 3, and Model 10. Points in each plot are fitted with a linear regression line (black) indicating the model's fit to the E-Sampler data. The models' performance is quantified by RMSE and MBE metrics displayed at the top of each plot.

FIGURE S5 6
Hourly-averaged $PM_{2.5}$ concentrations in April 2023, at the collocation site at Makerere University in Kampala. The dashed red line depicts the raw PurpleAir $PM_{2.5}$ concentration, solid black the Beta Attenuation Monitor from AirQo and the solid purple line represents the corrected PurpleAir concentration.

Figure S1. Time series comparison of hourly $PM_{2.5}$ concentrations measured by AirQo BAM and U.S. Embassy BAM devices from April 1st to April 18th, 2023. The x-axis represents the date, while the y-axis shows the $PM_{2.5}$ concentrations in micrograms per cubic meter (µg/m³). The green line represents the AirQo BAM measurements, and the black line represents the U.S. Embassy BAM measurements.

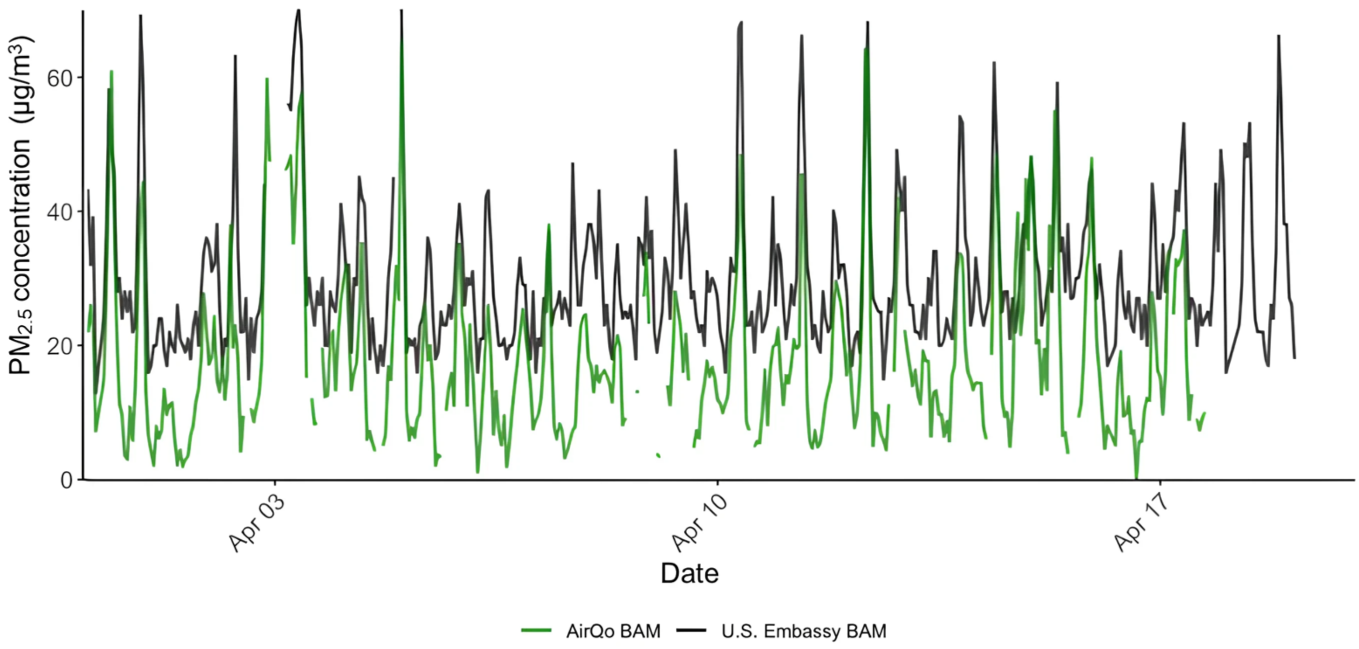

Figure S2. Scatter plot comparing the performance of three correction models against the E-Sampler hourly-averaged $PM_{2.5}$ concentration measurements. The x-axis represents the E-Sampler $PM_{2.5}$ concentrations in micrograms per cubic meter (µg/m³). The y-axis corresponds to the corrected PurpleAir $PM_{2.5}$ concentrations predicted by the Linear (Model 3), Random Forest, and XGBoost models. On each panel the red line represents the line of best fit. Spearman's rho quantifies the strength of the correlation between observed and predicted values.

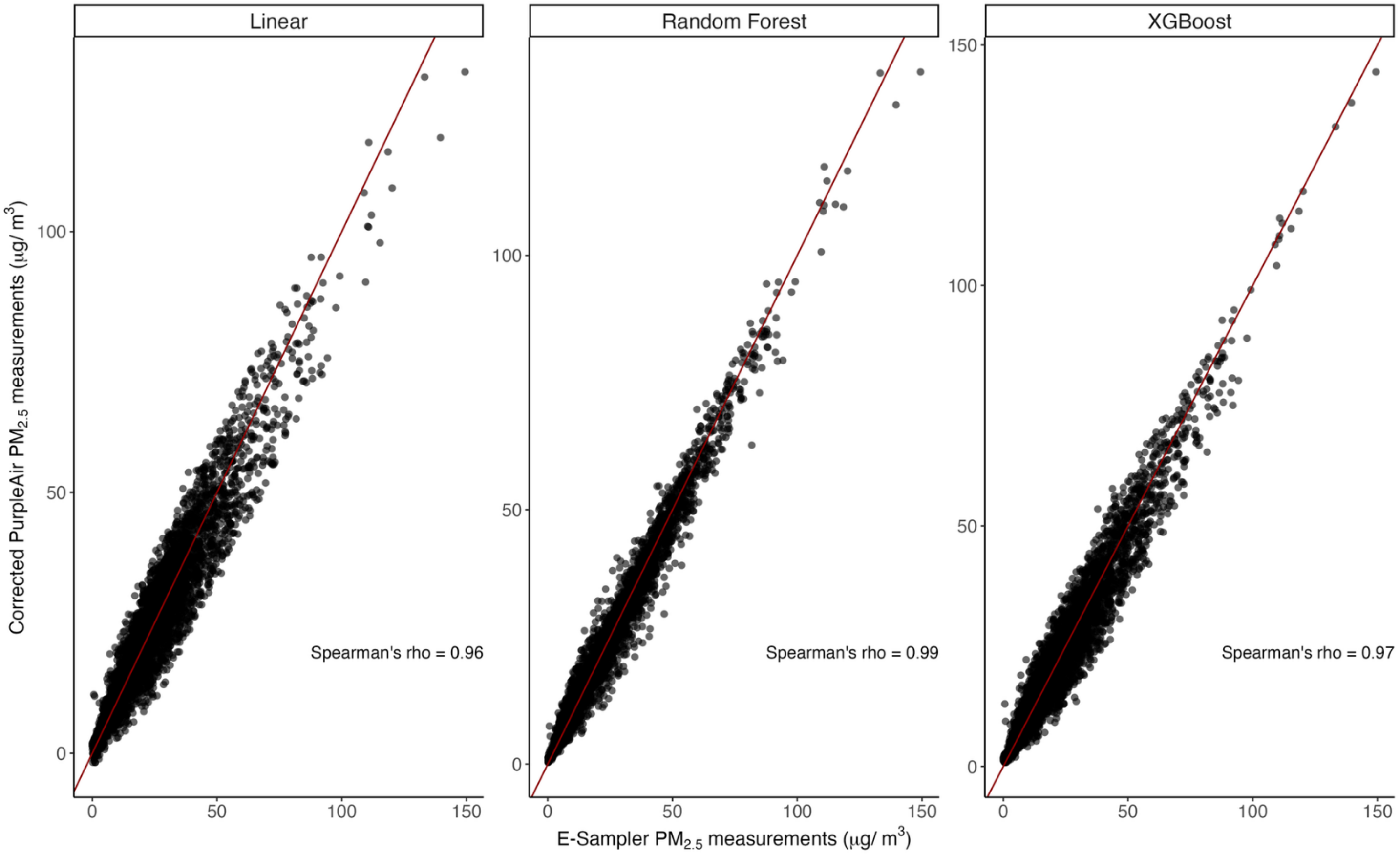

Figure S3. Bland-Altman plot of the agreement between the collocated corrected PurpleAir and E-Sampler hourly-averaged $PM_{2.5}$ concentration measurements at Kalisizo, Uganda. The x-axis displays the mean of the corrected PurpleAir and E-Sampler $PM_{2.5}$ concentrations). The y-axis shows the difference between the corrected PurpleAir and E-Sampler $PM_{2.5}$ concentrations. The solid horizontal line represents the mean difference (-0.07 μg/m³), and two dashed horizontal lines indicate the upper and lower limits of agreement (8.40 μg/m³ and -8.55 μg/m³, respectively). The blue line shows the trend in the bias across the range of measurements. The negative slope of the blue line suggests that the difference between the two methods increases as $PM_{2.5}$ concentration increases. This negative difference indicates that the corrected PurpleAir measurements are generally lower than the E-Sampler measurements at higher concentrations.

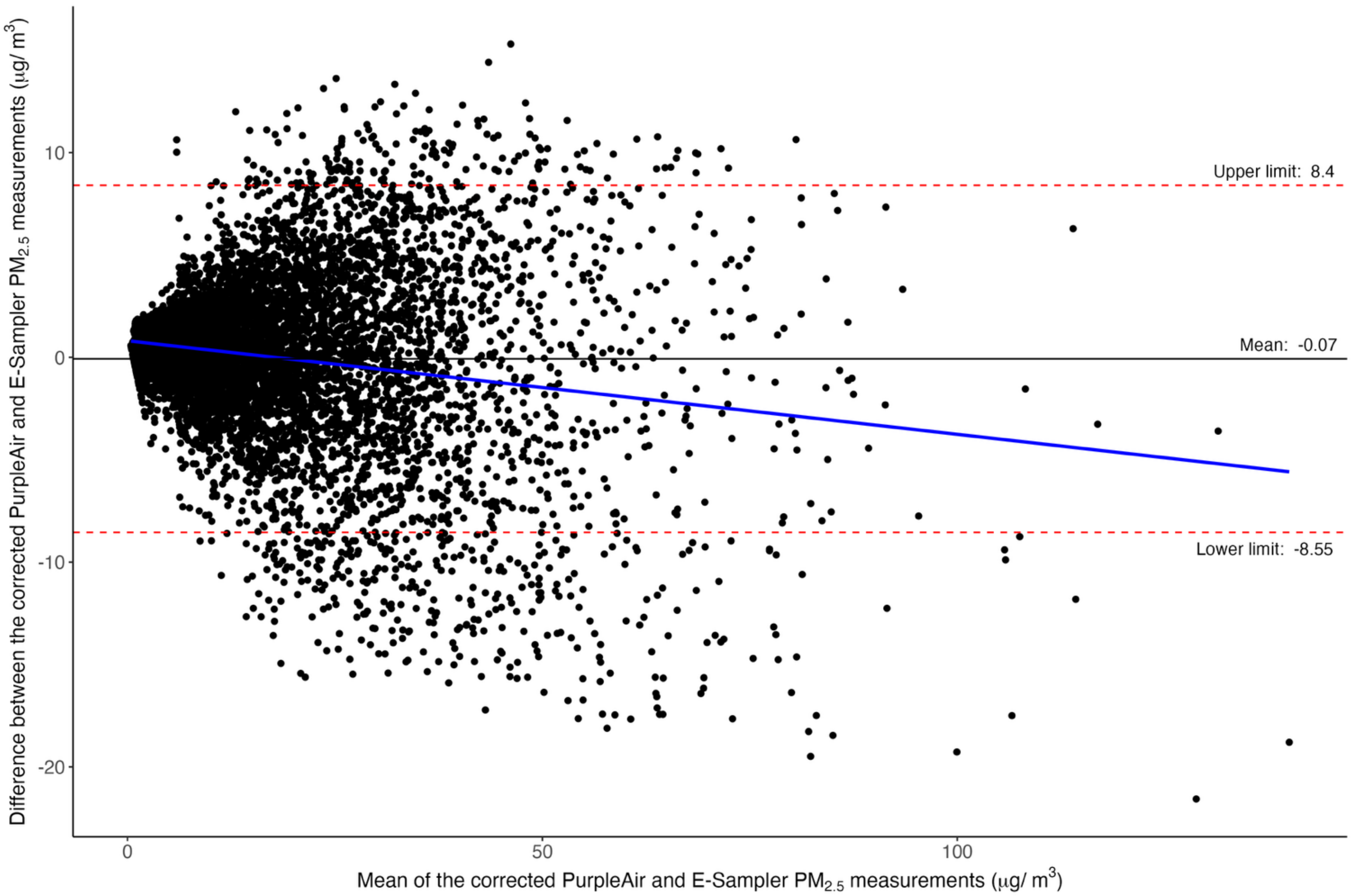

Figure S4. Comparative Analysis of $PM_{2.5}$ Concentrations Using Different Linear Calibration Models. This figure presents a comparison between corrected PurpleAir $PM_{2.5}$ concentrations using various models and the near reference grade E-Sampler measurements. Each plot aligns E-Sampler measurements (x-axis) against model predictions (y-axis) for Raw Data (note the different scale for the left panel), Model 1, Model 3, and Model 10. Points in each plot are fitted with a linear regression line (black) indicating the model's fit to the E-Sampler data. The models' performance is quantified by RMSE and bias metrics displayed at the top of each plot.

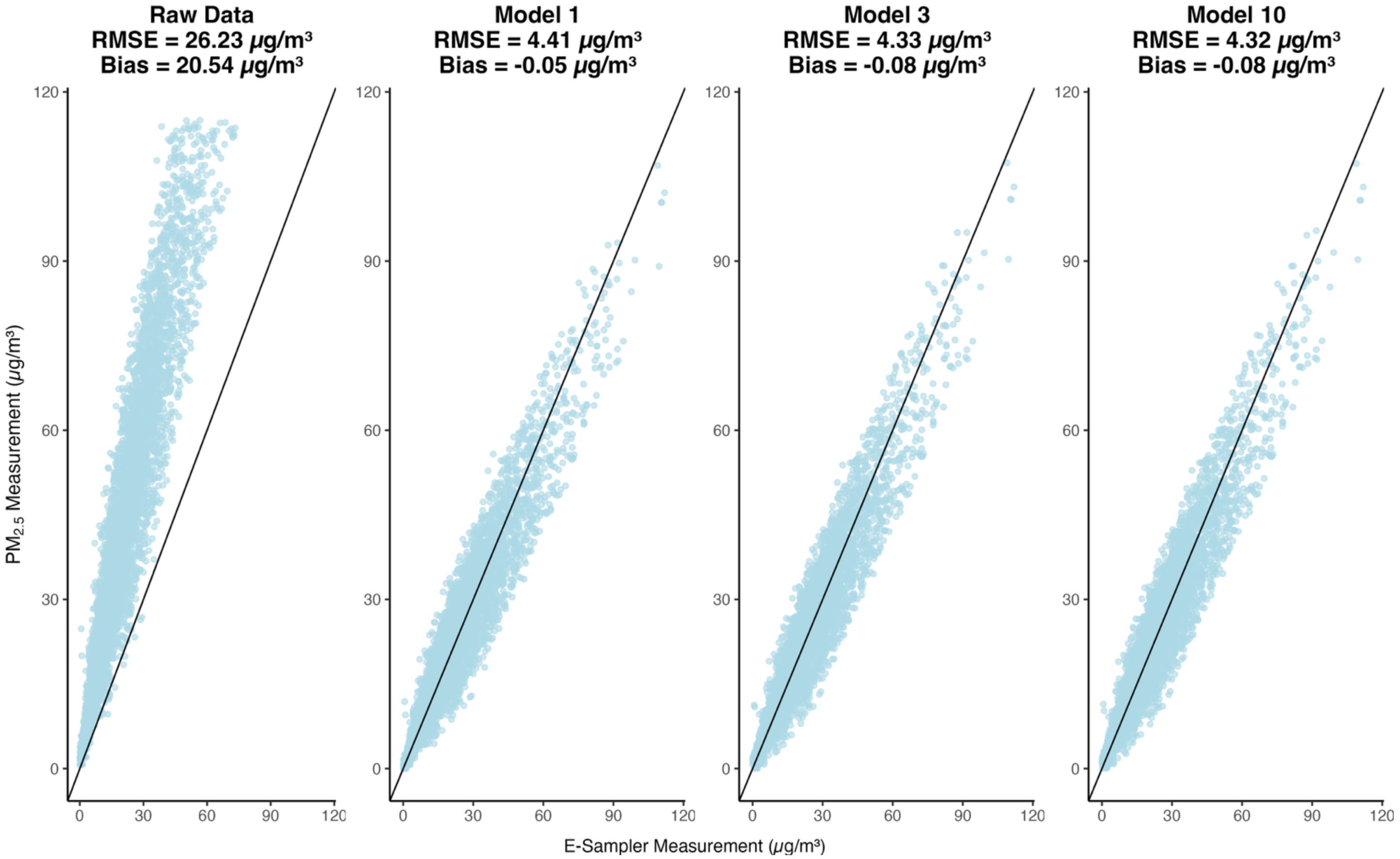

Figure S5. Hourly-averaged PM2.5 concentrations in April 2023, at the collocation site at Makerere University in Kampala. The dashed red line depicts the raw PurpleAir PM2.5 concentration, solid black the Beta Attenuation Monitor from AirQo and the solid purple line represents the corrected PurpleAir concentration.

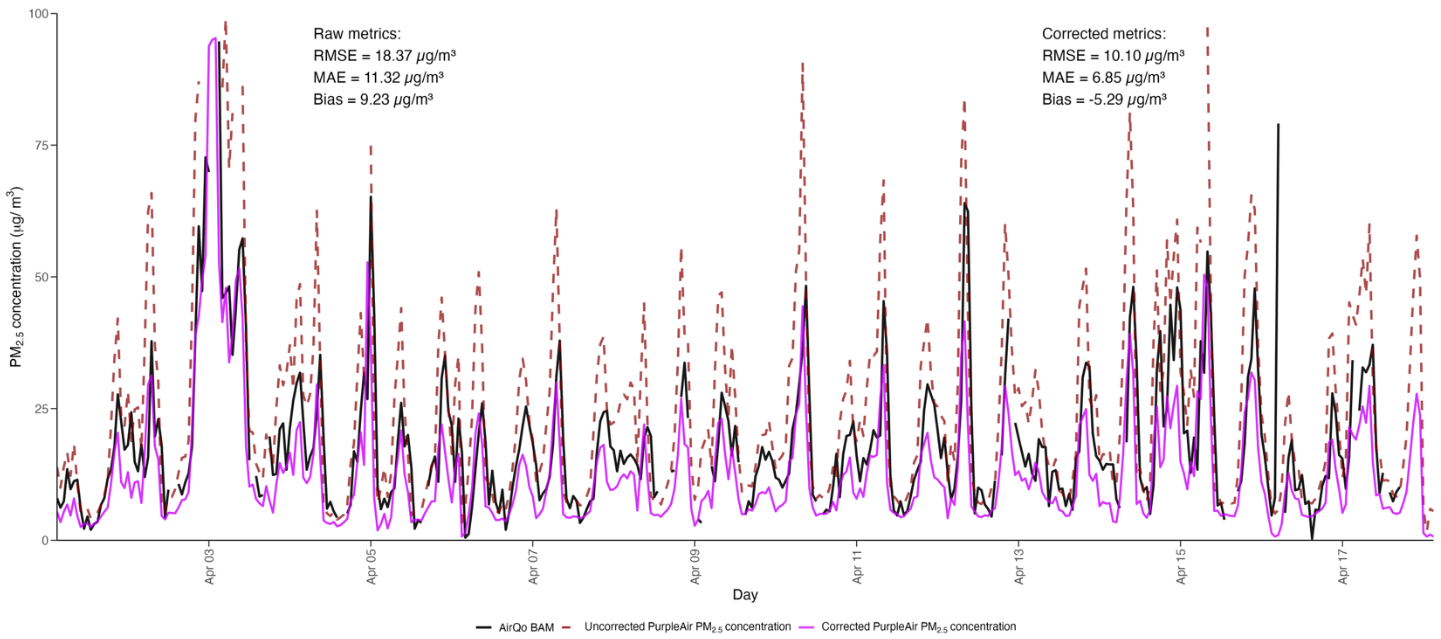

Supplement: Supplement [file NIHMS2088579-supplement-Supplement.pdf]
